# Rab13 regulates sEV secretion in mutant *KRAS* colorectal cancer cells

**DOI:** 10.1038/s41598-020-72503-8

**Published:** 2020-09-25

**Authors:** Scott A. Hinger, Jessica J. Abner, Jeffrey L. Franklin, Dennis K. Jeppesen, Robert J. Coffey, James G. Patton

**Affiliations:** 1 Department of Biological Sciences, Vanderbilt University Medical Center, Nashville, TN 37235 USA; 2 Department of Cell and Developmental Biology, Vanderbilt University Medical Center, Nashville, TN 37235 USA; 3 Department of Medicine, Vanderbilt University Medical Center, Nashville, TN 37235 USA; 4 Veterans Affairs Medical Center, Nashville, TN 37235 USA; 5 Vanderbilt University, Nashville, TN 37235 USA; 6 Present Address: Department of Physiology and Cell Biology, College of Medicine, The Ohio State University, Columbus, OH 43210 USA

**Keywords:** Colorectal cancer, Mechanisms of disease, Small GTPases

## Abstract

Small extracellular vesicles (sEVs), 50–150 nm in diameter, have been proposed to mediate cell–cell communication with important implications in tumor microenvironment interactions, tumor growth, and metastasis. We previously showed that mutant *KRAS* colorectal cancer (CRC) cells release sEVs containing Rab13 protein and mRNA. Previous work had shown that disruption of intracellular Rab13 trafficking inhibits epithelial cell proliferation and invasiveness. Here, we show that Rab13 additionally regulates the secretion of sEVs corresponding to both traditional exosomes and a novel subset of vesicles containing both β1-integrin and Rab13. We find that exposure of recipient cells to sEVs from KRAS mutant donor cells increases proliferation and tumorigenesis and that knockdown of Rab13 blocks these effects. Thus, Rab13 serves as both a cargo protein and as a regulator of sEV secretion. Our data support a model whereby Rab13 can mediate its effects on cell proliferation and invasiveness via autocrine and paracrine signaling.

## Introduction

Cell–cell signaling via extracellular vesicles (EVs) has been shown to play an important role in the development and progression of various cancers, with significant effects on proliferation, invasion and metastasis. Traditionally, EVs are classified both by size and biogenesis pathway^1^. Two major pathways have been described for EV release. In one, EVs are secreted by direct budding from the plasma membrane, including larger microvesicles (greater than 150 nm) and a heterogeneous mixture of smaller vesicles^2,3^. Smaller vesicles of endosomal origin (exosomes) are secreted when multivesicular bodies fuse with the plasma membrane releasing their intraluminal contents. These different classes of vesicles utilize functionally distinct mechanisms controlling cargo content with cell- and disease-specific effects^4–6^. However, it is now becoming clear that classifying vesicles into broad classes based on size is too simplistic as it ignores differences in cargo content and biogenesis. For these reasons, the preferred nomenclature is to refer to all secreted vesicles more broadly as EVs^7^ with differential centrifugation and density gradient centrifugation methods used to distinguish between small EVs, (sEVs), large EVs, and non-vesicular (NV) content^8^.

Previous work from our group using isogenic colorectal cancer cell lines has demonstrated that EV cargo content is regulated in a KRAS dependent manner, including proteins, miRNAs, circular RNAs, mRNAs, and long coding and non-coding RNAs (lncRNAs)^9–12^. We also showed that EVs can mediate functional transfer of cargo from donor to recipient cells. However, the cellular mechanisms by which specific proteins and RNAs are selected for secretion in EVs, as well as the biogenesis of the EVs themselves, remains to be fully elucidated.

Rab associated G proteins (Rabs) have been known for many years to play a role in regulating endocytic trafficking in mammalian cells^13^. Because some EVs are secreted through similar endocytic pathways, a regulatory role for Rab proteins in EV secretion was not unexpected. Indeed, Rab27a/b and Rab35 play key roles in the regulation of EV biogenesis^14,15^. We previously identified another Rab family member, Rab13, in EVs from mutant KRAS cells, but its precise role in EV secretion remains unclear^10^. Cellular Rab13 has been shown to play a significant role in cancer progression, invasiveness, and metastasis, partly related to the regulation of tight junctions and adherens junction formation^16–20^. Rab13 also regulates β1-integrin recycling in epithelial cells and β1-integrin has been shown to be a marker for EVs in multiple systems^17,21,22^. Further, β1-integrin + sEVs promote anchorage-independent growth in a pancreatic tumor model^23,24^. Here, we identify a novel and distinct subclass of β1-integrin+ /Rab13 + EVs, and furthermore, demonstrate that Rab13 regulates both the secretion of these new vesicles, as well as the secretion of classical exosomes.

## Results

### Rab13 regulates sEV secretion in a KRAS-dependent manner

**Figure 1:**
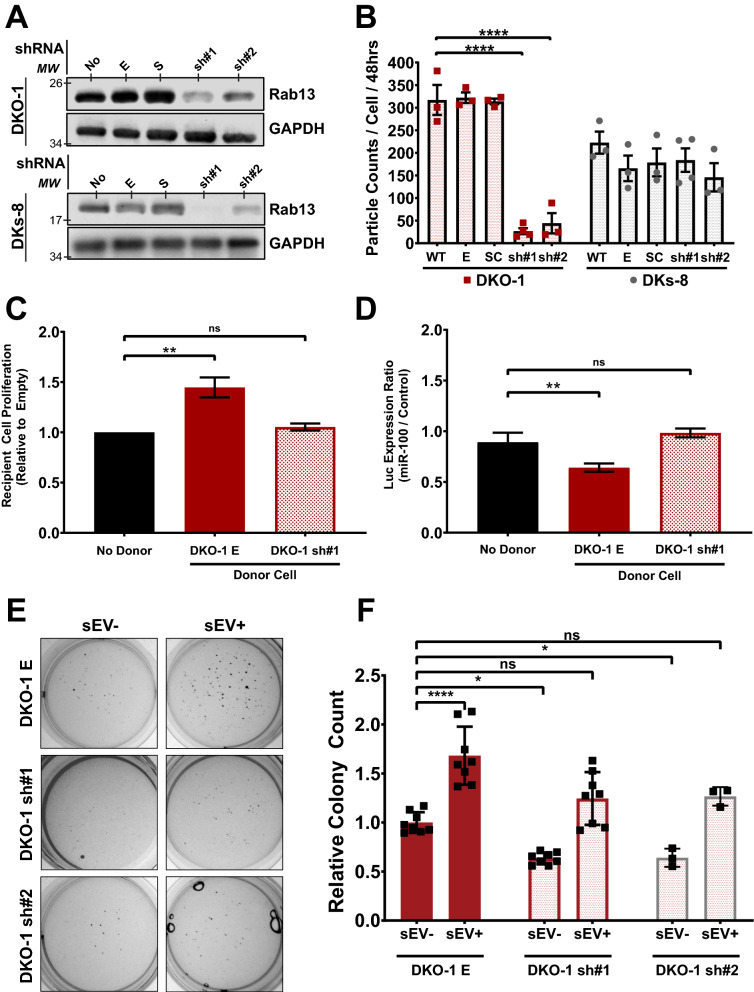
Rab13 regulates EV secretion in a KRAS-dependent manner. (**A**) Rab13 knockdowns. Stable lines expressing one of two independent shRNAs targeting Rab13 were created in wild type (DKs-8) and mutant (DKO-1) KRAS cells. Western blots were performed on cell lysates using antibodies against Rab13 or GAPDH on the parent cell lines (WT) or lines expressing an empty shRNA vector, a scrambled shRNA vector, or sh#1 or sh#2 against Rab13. (**B**) Nanosight-tracking analysis (NTA) of sEVs isolated from the cell lines outlined in (**A**). (**C**) Proliferation assays. DKs-8 recipient cells were co-cultured for 48 h in the presence of either no donor cells, donor DKO-1 cells expressing an empty shRNA vector (**E**), or donor DKO-1 cells stably expressing sh#1. Recipient cells were then collected and total cells were counted and plotted relative to the number of cells counted in the absence of any donor cells. (**D**) Luciferase reporter assays. DKs-8 recipient cells were transiently transfected with a vector expressing luciferase fused to a control 3′ UTR or a 3′UTR containing three perfect binding sites for *miR-100*. Recipient cells were then co-cultured for 48 h with either no donor cells, donor DKO-1 cells expressing an empty shRNA vector (**E**), or donor DKO-1 cells stably expressing sh#1. Cell lysates were prepared and luciferase expression was measured with decreased luciferase expression representing more transfer of *miR-100*. (**E**) DKO-1 cells with or without Rab13 knockdown were grown in soft agar for 2 weeks in the presence or absence of sEVs purified from DKO-1 cells. (**F**) Quantification of colony counts from three biological soft agar assay replicates. Significance was determined by one-way ANOVA. *p < 0.05, **p < 0.01, ****p < 0.0001. Data represent mean ± SE, n = 3. *Ns* no significance.

We previously showed that Rab13 mRNA and protein are enriched in sEVs from KRAS-mutant CRC cells^10^. Given proposed functional roles for Rab13, we hypothesized that Rab13 might regulate the secretion of sEVs. Independent stable KRAS-mutant and KRAS-wildtype CRC cells were generated that express shRNAs against Rab13 mRNA leading to a 70–80% reduction in Rab13 protein between the two distinct shRNA lines (Fig. 1A). Conditioned media were collected from control and knockdown cells and crude sEVs were purified. Particle counts were analyzed by nanoparticle tracking analysis (Supplementary Fig. S1) and quantified relative to input cell counts. We observed a dramatic decrease in particle counts/cell when Rab13 was knocked down in two different mutant KRAS DKO-1 cell lines, but not after knockdown in wild type KRAS DKs-8 cells (Fig. 1B). Despite the decrease in sEVs, we did not observe any defects in cell proliferation (Supplementary Fig. S2), in contrast to previous work using breast cancer epithelial cells^18^.

To test the functional significance of Rab13-dependent secretion of sEVs, we utilized two distinct Transwell assays (Supplementary Fig. S3a). We first tested whether parental DKO-1 cells, which express normal levels of Rab13, would affect the proliferation of DKs-8 cells in a co-culture environment. When plated on opposite sides of a Transwell membrane, the presence of DKO-1 cells in the donor compartment increased the proliferation of recipient DKs-8 cells when compared to control conditions with no donor cells (Fig. 1C). However, Rab13 knockdown in the donor DKO-1 cells abrogated the effect on proliferation in DKs-8 recipient cells following co-culture (Fig. 1C). Knockdown of Rab13 in KRAS wildtype DKs-8 donor cells did not affect recipient cell proliferation (Supplementary Fig. S3b). This suggests that Rab13 regulates extracellular secretion in KRAS mutant CRC cells and that decreased levels of Rab13 blocks proliferation inducing effects in recipient cells.

Previously, we showed that *miR-100* can undergo functional transfer from donor mutant KRAS cells to recipient wild type KRAS cells using luciferase reporter assays^25^. When we tested whether Rab13 knockdown might alter *miR-100* transfer, we again found that decreased levels of Rab13 reduced extracellular transfer of *miR-100* from DKO-1 donor to DKs-8 recipient cells resulting in increased luciferase reporter expression (Fig. 1D).

### Rab13 regulates anchorage-independent growth via sEVs

sEVs from mutant KRAS cells can promote proliferation and anchorage-independent growth in wild type KRAS cells^9,26^. Thus, we tested whether Rab13 knockdown in DKO-1 cells would alter colony formation in soft agar assays. First, DKO-1 cells were incubated with DKO-1 sEVs prior to embedding in soft agar. Exposure of DKO-1 cells to sEVs in this manner increased the total number of colonies in soft agar, compared to untreated DKO-1 cells, consistent with previous results^9^ (Fig. 1E,F). We then tested the effects of Rab13 knockdown on colony growth in soft agar. Loss of Rab13 slightly reduced the number of colonies in soft agar (Fig. 1F). However, exposure of the knockdown lines to sEVs from normal DKO-1 cells restored the colony counts back to control DKO-1 levels (Fig. 1F). Besides growth in soft agar, we also found that sEVs from DKO-1 cells caused an increase in tumor-like, migratory colonies when grown in type-1 collagen (Supplementary Fig. S4). Again, knockdown of Rab13 blocked this effect, whereas exposure to sEVs from DKO-1 cells rescued migratory colony numbers (Supplementary Fig. S4). Together, these results suggest that Rab13 regulates anchorage-independent and 3D growth through regulation of sEV secretion, linking sEV biogenesis and tumorigenesis in colorectal cancer.

Although knockdown of Rab13 caused an 80–90% decrease in particle counts/cell (Fig. 1B), we nevertheless sought to test whether EVs from knockdown cells could rescue colony counts as in Fig. 1E,F. For this, we scaled up to generate equivalent amounts of vesicle preparations from knockdown cells and exposed DKO-1 cells to those EVs. As shown in Supplementary Fig. S5, we found that EVs from Rab13 knockdown cells could increase the total number of colonies in DKO-1 cells and rescue colony growth in knockdown cells in soft agar. This indicates that even though Rab13 knockdown dramatically reduces EV release, the resulting EVs still contain cargo that can promote cell proliferation.

### Rab13 regulates the secretion of sEV markers

**Figure 2:**
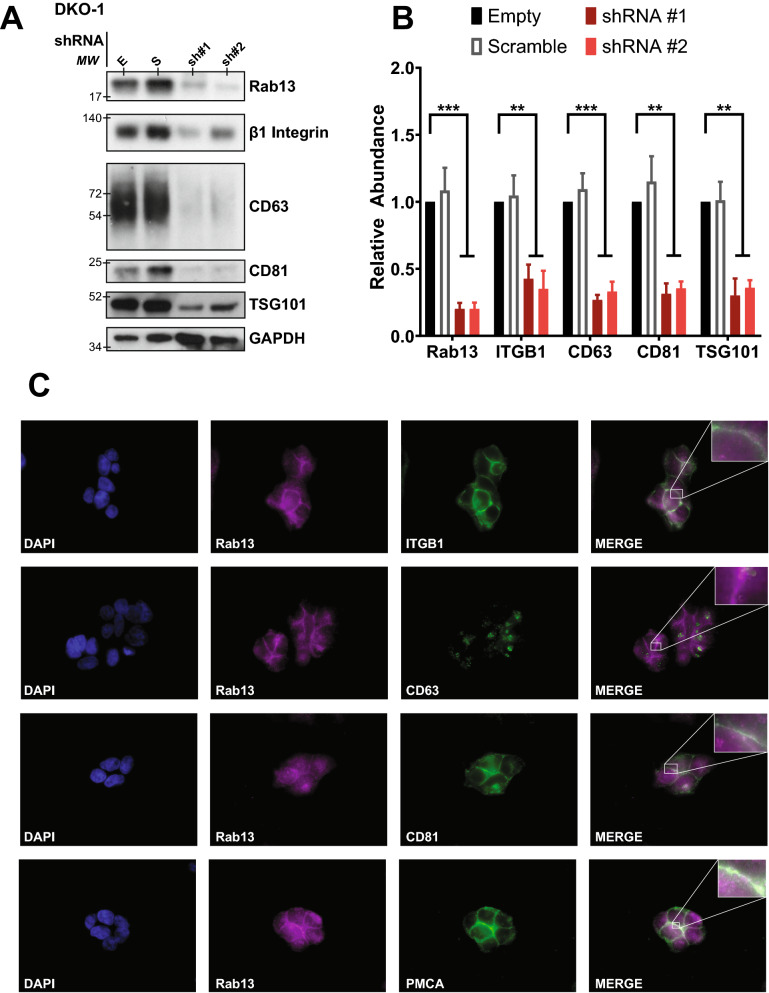
Rab13 regulates and co-localizes with sEV markers. (**A**) Cell lysates prepared from DKO-1 cells expressing an empty shRNA vector (E), a scrambled shRNA vector (S), or shRNAs targeting Rab13. Western blots were performed with antibodies against β1-integrin, CD63, CD81, and TSG101. (**B**) Quantification of immunoblots (n = 3) of sEV markers under Rab13 knockdown conditions. (**C**) Immunolocalization of Rab13, sEV and plasma membrane (plasma membrane Ca2+ ATPase, PMCA) markers in DKO-1 cells. Cells were stained with DAPI (blue), Rab13 (magenta), or antibodies against β1-integrin, CD63, CD81, or PMCA (green). Rab13 co-localized with the plasma membrane when cell–cell contact was made between neighboring cells. Significance was analyzed by one way ANOVA. **p < 0.01, ***p < 0.001. Data represent mean ± SE, n = 3.

To further investigate the role that Rab13 plays in vesicle secretion, sEVs were isolated from control and knockdown cells (Fig. 2A). Consistent with the overall decrease in sEVs observed after knockdown of Rab13, we observed reduced levels of the classical exosome markers CD63, CD81, and TSG101 (Fig. 2B). Previous work has shown that β1-integrin can be detected in EVs^27,28^ and that Rab13 can regulate β1-integrin recycling in an epithelial cancer model^17^. Furthermore, β1-integrin was detected in non-exosomal EVs^8^. Thus, we tested whether knockdown of Rab13 would reduce secretion of β1-integrin. As shown in Fig. 2A,B, knockdown of Rab13 reduced the levels of secreted β1-integrin.

### Rab13 and β1-integrin co-localize at the plasma membrane

Integrins belong to a family of cell adhesion receptors consisting of heterodimers with α and β transmembrane subunits that interact with the extracellular matrix. Integrins have been implicated in cancer progression and metastasis^29–32^. Tumor cells express distinct subsets of integrins and it has been proposed that exosomal integrins can predict organ-specific metastases^31,33^. In most studies examining integrins as EV cargo, the distribution of integrins in EV subclasses, including classical exosomes, is complex^8^, depending on the exact integrin interrogated. Rab13 has been implicated in β1-integrin trafficking to the cell surface^17^, but its role in EV trafficking remains unknown. To gain insight into the mechanisms regulating secretion of Rab13 and β1-integrin, DKO-1 cells were fixed and stained for endosomal and plasma membrane markers. Rab13 and β1-integrin were found to co-localize primarily at the plasma membrane in DKO-1 cells with little to no co-localization with CD63, a marker of the MVB and of classical exosomes (Fig. 2C). In contrast, Rab13 and β1-integrin were found to co-localize at the plasma membrane with CD81, another classical exosome maker (Fig. 2C).

### β1-integrin is enriched in EVs

**Figure 3:**
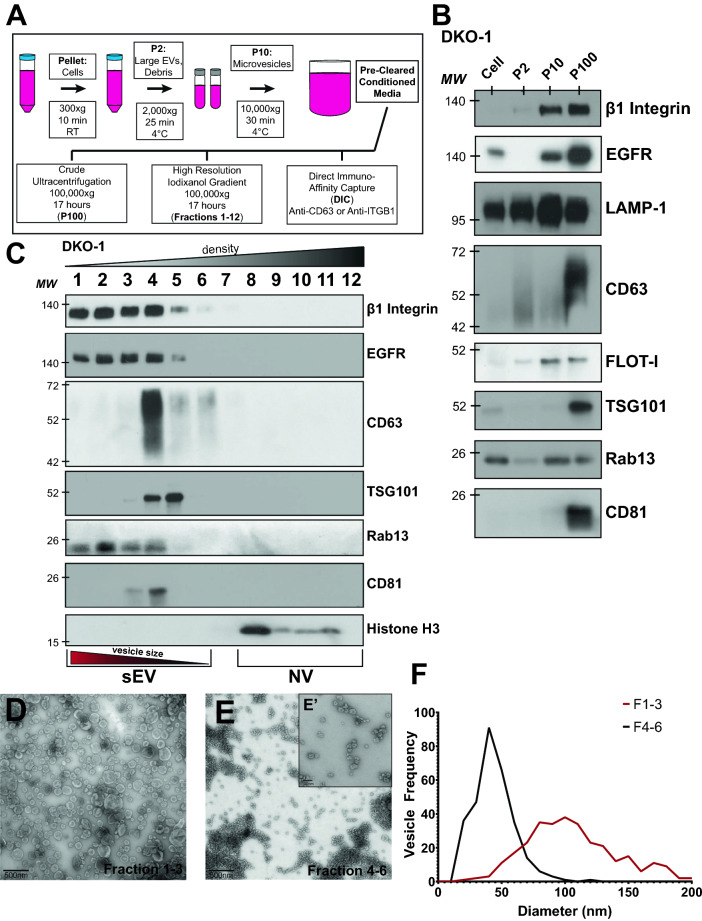
Identification of β1-integrin+, Rab13 + sEVs. (**A**) sEV purification protocol. Conditioned media were collected from DKO-1 cells and subjected to differential centrifugation to pellet cells, large EVs, cell debris, and microvesicles, as indicated. Pre-cleared conditioned media was then subjected to one of three different isolation techniques: overnight ultracentrifugation at 100,00×*g* (crude sEVs), high resolution iodixanol gradient purification, or direct immuno-affinity capture. (**B**) Fractions described in (**A**) were subjected to western blot analysis using antibodies against the indicated proteins. (**C**) Immunoblots of sEVs and non-vesicular (NV) fractions purified from DKO-1 cells following high resolution 12–36% iodixanol gradient purification. (**D**) Transmission electron microscopy (TEM) of vesicles isolated by high resolution iodixanol gradients, fractions 1–3. Scale bar 500 nm. (**E**) Transmission electron microscopy (TEM) of vesicles isolated by high resolution iodixanol gradient purification, fractions 4–6. Scale bar = 500 nm. (**E**’) Higher resolution TEM image from (**E**) Scale bar 100 nm. (**F**) Vesicle sizes from the high resolution iodixanol gradients were determined and plotted. Black line corresponds to fractions 4–6 and red line corresponds to fractions 1–3. The mean diameter of the vesicles in fractions 1–3 was significantly different (p value = 2.4 × 10^–79^) from the mean diameter in fractions 4–6 by Student’s t test.

To identify which specific class of EVs contain β1-integrin, media was collected from CRC cells and crude large EVs (P2), microvesicles (P10), and sEVs (P100) were purified from KRAS mutant DKO-1 cells by differential centrifugation (Fig. 3A). Using this method, β1-integrin and Rab13 were both detected in the P10 and P100 samples, which include microvesicles and sEVs, respectively (Fig. 3B). This is distinct from CD63, which was detected primarily in the P100 samples (Fig. 3B).

To test whether enrichment of β1-integrin and Rab13 in the P100 fractions is vesicle associated or not, we utilized ultracentrifugation followed by high resolution iodixanol density gradients^8^. Using this method, Rab13 and β1-integrin were found to co-sediment in fractions 1–3 which correspond to sEVs (Fig. 3C). Non-vesicular protein aggregates localize to the higher density fractions in these gradients^8^. Rab13 and β1-integrin were more enriched in the lighter density fractions (fractions 1–3) compared to fractions 4–6 which are enriched in the classical exosome markers CD63 and TSG101, and when compared to the non-vesicular (NV) fractions (9–12) which are enriched in histone H3. This suggests that Rab13 and β1-integrin are associated with vesicles that are larger in size and/or more lipid rich, compared to classical exosomes. Transmission electron microscopy (TEM) images on vesicles purified from either fractions 1–3 or 4–6 showed a clear difference in vesicle size, indicating that this population of vesicles is unique from classical exosomes (Fig. 3D–F). The iodixanol gradients show that sEVs enriched in β1-integrin are more heterogeneous in size, as they were detected not only in the P10 pellet (microvesicles), but also across a range of sEV sizes and densities (Fig. 3B,C). These data suggest that β1-integrin + EVs are distinct from classical CD63 + exosomes and are both Rab13-associated and Rab13-dependent for biogenesis.

EGFR was also enriched in light fractions 1–3, co-sedimenting with both Rab13 and β1-integrin, suggesting the potential for EGFR+, β1-integrin+, Rab13+ sEVs (Fig. 3C). Rab13 has been shown to regulate both EGFR and β1-integrin recycling to the plasma membrane, further supporting that these vesicles are likely PM-derived^17^. β1-integrin has been linked to the tumorigenic properties of EGFR in both lung and breast cancer and Rab13 may mediate this interaction, both intra- and extracellularly^34,35^. SNARE-dependent association of β1-integrin and EGFR association can regulate invadopodia formation and tumor cell invasion in a process that might be driven, in part, by EV-mediated mechanisms^36^. Together, this suggests that β1-integrin+, EGFR+, Rab13+ sEVs are secreted by KRAS-mutant cancer cells, and that these vesicles play important roles in cancer development and progression.

### β1-integrin + EVs are distinct from CD63+ exosomes

**Figure 4:**
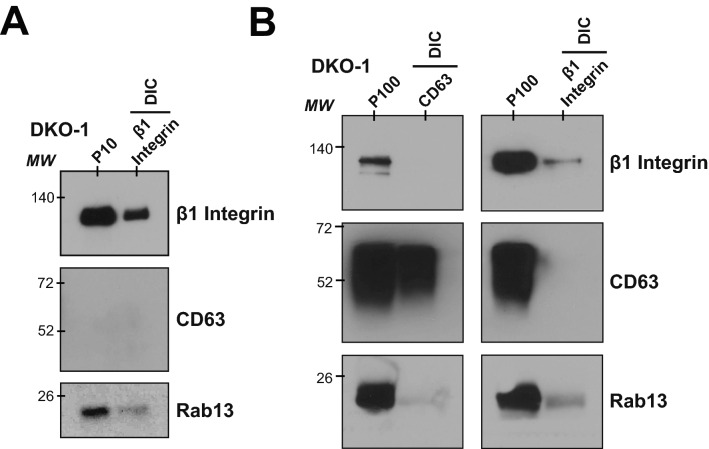
β1-integrin+/Rab13+ EVs are distinct from CD63+ exosomes. Direct immunoaffinity capture (DIC) was used to purify vesicles associated with either β1-integrin or CD63^8^. (**A**) Vesicles from the P10 centrifugation step were immunoaffinity captured with antibodies against β1-integrin and western blots were performed on the captured material using antibodies against β1-integrin, CD63 or Rab13. (**B**) Vesicles from the P100 centrifugation step were immunoaffinity captured with antibodies against either CD63 or β1-integrin and western blots were performed as in A. DIC shows a distinction between β1-integrin + sEVs and CD63+ classical exosomes, with an enrichment of Rab13 in the β1-integrin + sEVs.

To further test if β1-integrin + EVs are distinct from CD63 + classical exosomes, we used direct immuno-affinity capture (DIC) to isolate distinct EVs enriched in both the P10 microvesicle and P100 sEV samples^8^. P10 (Fig. 4A) or P100 (Fig. 4B) samples were incubated with beads coated with antibodies against either β1-integrin or CD63. After incubation, the beads were extensively washed, the bound material was released in lysis buffer, and western blots were performed. When beads coupled to β1-integrin were incubated with P10 fractions, the bound material was enriched for both β1-integrin (as expected) and Rab13, supporting the hypothesis that these proteins are in the same vesicle (Fig. 4A). Due to low abundance of CD63 in the P10 microvesicle population, DIC against CD63 was unsuccessful. Instead, DIC was carried out on P100 samples using beads coated with antibodies against CD63+ (classical exosomes) or β1-integrin. With this method, no association was detected between CD63 and β1-integrin (Fig. 4B). Analysis of the P100 fractions showed enrichment of Rab13 in β1-integrin + sEVs, further confirming the link between Rab13 and β1-integrin + EVs (Fig. 4B).

Rab13 was also detected in fractions overlapping with CD63+ (classical exosomes), which could explain why knockdown of Rab13 also reduced the secretion of classical exosome markers (Fig. 2), although Rab13 might also affect biogenesis of EVs without itself being a cargo protein. Together, the data support a novel population of β1-integrin + EVs distinct from classical exosomes that are enriched in both the P10 microvesicle population and the P100 sEV population. The data also demonstrate a link between Rab13 and β1-integrin + EVs, supporting a new population of EVs whose biogenesis is Rab13-dependent.

## Discussion

### Rab13 regulates EV secretion in mutant KRAS cells

In this study, we demonstrate that Rab13 can regulate the secretion of sEVs in a KRAS-dependent mechanism. Previous work has shown that Rab27a/b and Rab35 regulate EV secretion^14,15^. Our work expands the number of Rab family members that can regulate EV biogenesis and also uncovers a potential new mechanism of secretion distinct from fusion of MVBs with the plasma membrane. Rab proteins as a family may play a significant role in the secretion of multiple subclasses of EVs, with the caveat that the specific role played by individual Rab proteins may be both cell-type and disease-state specific.

Notably, we did not observe any effects on sEV secretion after knockdown of Rab13 in wild type KRAS cells (Fig. 1B). Also, when we measured proliferation in Transwell assays, we did not observe any effects on recipient cells when Rab13 was knocked down in wild type KRAS donor cells (Supplementary Fig. S3B). This indicates that signaling pathways downstream of constitutively active KRAS are necessary for the effects we observe after knockdown of Rab13 that could be similar in other cancer cells with KRAS mutations. One possibility is that DENND2B, a known GEF of Rab13, activates Rab13 via regulation by KRAS signaling^18^. DENND2B is down regulated by protein kinase D (PKD)^18,37^ which is transcriptionally regulated by the KRAS-NF-κB signaling cascade in pancreatic cancer, suggesting that Rab13 may be functioning in a DENND2B-independent mechanism when regulating EV secretion in KRAS-mutant cells^38^. Further understanding of how KRAS activation drives Rab13-dependent EV secretion is required to determine the cellular contexts in which these EVs are regulated and released, and whether the affects we observe extend beyond colorectal cancer.

Rab13-dependent trafficking of β1-integrin has been shown in breast cancer epithelial cells^17^ and β1-integrins have been identified in sEVs released from a number of cells, notably breast and pancreatic cancer cells^27,31,33^. We show that Rab13-dependent sEVs promote cell growth, miRNA transfer, and anchorage-independent growth in a KRAS-dependent manner in CRC cells. Decreased levels of Rab13 reduced secreted β1-integrin levels and inhibited cell growth, miRNA transfer, and anchorage-independent growth. Previously, classical exosomes containing β1-integrin were proposed to promote anchorage-independent growth in a pancreatic tumor model^23^. By immunocapture, we observed few, if any, β1-integrin-containing vesicles associated with the classical exosome marker CD63 suggesting that β1-integrin+, Rab13-dependent sEVs might be responsible for promoting anchorage-independent growth.

### Rab13 localization and cell confluency

Our immunostaining data (Fig. 3) shows that Rab13 localizes to the plasma membrane and co-localizes with β1-integrin in mutant KRAS cells. We performed the localization experiments at the same culture times and conditions as when we isolated EVs. However, in the course of those experiments, we noticed that when cells are plated at low density, Rab13 localizes more broadly to the cytoplasm and then re-localizes to the plasma membrane when cell–cell contact occurs. It is possible that the role that we observe for Rab13 in regulating EV secretion, especially secretion of β1-integrin+, Rab13+ sEVs, is dependent on culture density or confluency. Interestingly, we previously observed differences in miRNA secretion between mutant (DKO-1) and wild type (Dks-8) KRAS cells^11,39^. Even when cultured at low density, mutant KRAS cells tend to clump together and make contact with one another, whereas individual wild type KRAS cells spread out and only come into contact as the confluency increases. The secretion of β1-integrin+, Rab13+ vesicles (and possibly other EVs) might be confluency-dependent which could also account for differences in miRNA export.

### Heterogeneity in sEV populations

There is growing evidence that there is significant EV heterogeneity, even among traditional subclasses such as exosomes. Although exosomes have traditionally been described as the primary form of sEVs, not all vesicles ~ 40 to 150 nm are classical exosomes^8^. Classifying EV populations by size is not sufficient to characterize or identify one subclass over another. Full understanding of overall EV heterogeneity requires standardized purification methods^40^, as well as an appreciation that EV cargo can differ in a cell-context manner. The recent use of high resolution density gradient fractionation methods further demonstrates that the exact purification strategies adopted can also affect the presence of protein and RNA cargo^8^. When we employed high resolution gradients, we discovered that Rab13 is associated with β1-integrins in a population of sEVs that are distinct from classical CD63+ exosomes. Furthermore, we found that Rab13 is required for the efficient release of EVs in mutant KRAS cells but not in isogenic wild type KRAS cells. β1-integrin+ EVs are found in both P100 sEV and P10 microvesicle fractions, unlike classical CD63+ exosomes which are enriched in P100 sEV populations. This suggests that there may be commonalities between what are currently considered classical sEVs and classical microvesicles. The cellular localization and size of β1-integrin+/Rab13+sEVs suggest that these EVs are more likely secreted from the plasma membrane than via fusion of MVBs with the plasma membrane.

The high resolution density gradients that we utilized followed a slightly different protocol from ****Jeppesen et al. (2019) with differences in ultracentrifugation times and we did not subject supernatants from conditioned media (see Fig. 4) to filtration through a 0.2 μm filter before the density gradients. Had we followed the exact protocol described by Jeppesen et al.^8^, the larger β1-integrin+, Rab13+ vesicles would not have been present in the gradients and therefore would not have been detected. This further emphasizes the point that the extent of vesicle heterogeneity is dependent on the choice of purification protocol. Our results expand what is known about EV heterogeneity and biogenesis, adding a new wrinkle that EV cargo composition across different EV subclasses can depend on Rab13 and KRAS status.

## Methods

### Cell culture

DKO-1 and DKs-8 colorectal cancer cells are isogenic cell lines derived from the parental DLD-1 line^41^. DLD-1 cells are heterozygous for KRAS, Dks-8 cells contain a single wild type allele of KRAS, and DKO-1 cells contain a single mutant KRAS allele. Cells were cultured in standard DMEM (Glibco) with 10% FBS, 1% non-essential amino acids, 1% l-glutamine, and 1% penicillin/streptomycin (Glibco). Cells were grown at 37 °C in 5% CO_2_. Cells were passaged a maximum of 10 times before being discarded.

### Isolation of EVs

EVs were isolated through three varying levels of purity. Cells were seeded into 3–20 T175 flasks (Corning) at a density of 6.5 × 10^6^ (DKO-1) or 7.5 × 10^6^ (DKs-8) cells per flask. Cells were grown in the presence of serum to 80% confluency (~ 48 h), washed three times with 1 × Dulbecco’s PBS (DPBS; Gilbco), and then grown for 48 h in serum free media. Cell-conditioned media (CM) was collected and subjected to differential centrifugation in three steps: 300×*g* for 10 min (room temperature), 2000×*g* for 25 min (4 °C), and then 10,000×*g* for 30 min (4 °C). These steps produce cell pellets, cell debris and large EVs, and microvesicles, respectively. P100 pellets (crude sEVs) were obtained by centrifuging CM that had been subject to the three steps above for additional 17 h at 100,000×*g* (4 °C). Pellets were suspended in 1 × PBS and washed by centrifugation at 100,000×*g* twice for 70 min each time (4 °C). Pellets were then resuspended in 20 µL 1 × DPBS or 1 × RIPA lysis solution. For gradient preparations, a modified version of the protocol used in Jeppesen et al. 2019 was used^8^. Briefly, CM subject to the three steps above was concentrated using a 100 K concentrator (MilliPore) down to ~ 5 mL. The concentrated media was centrifuged at 100,000×*g* for 17 h (4 °C). The pellet was then resuspended in 2.4 mL of 36% iodixanol solution (Optiprep) and then transferred to a new tube. A chilled iodixanol discontinuous gradient consisting of 2.4 mL layers of 30%, 24%, 18%, and 12% iodixanol were layered on top of the 36% iodixanol solution containing the sEVs. After centrifugation at 100,000×*g* for 17 h (4 °C), 1 mL fractions (12) were collected (top to bottom). Each fraction was diluted in 10 mL 1 × DPBS and then centrifuged at 100,000×*g* for 3 h (4 °C). Final pellets were resuspended in 10–30µL 1 × DPBS or 1 × RIPA lysis buffer for western blots.

### Nanoparticle tracking analysis (NTA)

Particle sizes and numbers were analyzed using the Zetaview^®^ Nanoparticle Tracking Video Microscope PMX-120 (Particle Matrix) and associated software. After optimization of the software, settings were held constant across all four samples for each replicate. Typical concentration of vesicles ranged from 10^8^ to 10^11^ particles/mL.

### Direct immunoaffinity capture of sEVs

DIC was carried out as previously published^8^. In short, antibodies against either CD63+ (BD bioscience) or β1-integrin+ (BD bioscience) were bound to Dynabeads (Thermofisher) at 5 μg of antibody per 1 mg beads. Conjugated beads were then washed, and incubated with DKO-1 pre-cleared conditioned media overnight, agitating at 4 °C. Following incubation, beads were washed three times in DIC wash buffer, then re-suspended in 1 × RIPA buffer (ThermoFisher). Samples were then loaded onto PAGE gels (Biorad) for western blot analyses.

DIC against the P10 pellet (microvesicles) was carried out in the same fashion but the P10 pellet was resuspended in DIC wash buffer, and then incubated with conjugated beads.

### Plasmid construction

Human Rab13 cDNA was synthesized using Accuscript RT (Agilent Technologies) from RNA purified from DKO-1 CRC cells. Rab13HA cDNAs were inserted into the pcDNA3.1 + (Zeocin) plasmid using BamHI and NotI (New England Biolabs). shRNA plasmids were a gift from the Vanderbilt shRNA Core (Vanderbilt University). Rab13 shRNA #1 targeted the 3′UTR while shRNA #2 targeted the 3′ end of the Rab13 ORF. All plasmids were confirmed by sequencing (Genewiz, South Plainfield, NJ, USA).

### Primers

Rab13HA_F: 5′-ATACGGATCCATGCCAAAGCCTACGAC.

Rab13HA_R: 5′- ATACGCGGCCGCTCAAGCGTAATCTGGAACATC.

### Protein collection and western blotting

Protein concentrations were quantified by BCA assays (BIO-RAD). For western blots, proteins were denatured in 1 × RIPA buffer (Life Technologies). 2–5 μg of total protein were loaded onto pre-cast gels (10 or 12% MINI-PROTEAN TGX^®^ 12 by 15 or 50 μL well pre-casted gel, respectively; BIO-RAD). Separated proteins were transferred to nitrocellulose using the Trans-Blot^®^ Turbo Transfer System (BIO-RAD). Membranes were blocked in 5% milk in TBS-T for 1 h at room temperature. Primary antibodies were incubated overnight in 5% milk in TBS-T at 4 °C. Secondary antibodies were incubated in 5% milk in TBS-T for 1 h at room temperature. Blots were then washed in 1 × TBS-T three times. For visualization, membranes were treated with SuperSignal™ West Femto Maximum Sensitivity Substrate for 60 s (Thermo Scientific). Blots were then exposed to film and developed for ~ 1 s to ~ 5 min depending on band intensity.

### Transfection of shRNA plasmids and Rab13HA expression plasmids

Plasmids were transfected using the Lipofectamine 2000 protocol (ThermoFisher). Cells were seeded at 0.1 × 10^6^ cells per well in 12 well dishes (Corning) and incubated for 24 h at 37 °C. 500–1000 ng of DNA were incubated with 5 µL Lipofectamine 2000 per well for 20 min at room temperature, and then added to individual wells. Cells were then incubated at 37 °C for 24 h before antibiotic selection for 1–2 weeks. shRNA vectors were selected using 1 µg/mL Puromycin while Rab13HA expression vectors were selected using 50–100 mg/mL Zeocin (ThermoFisher). shRNA transfections were confirmed via GFP visualization and Rab13HA expression was confirmed by western blot detection via an α-HA antibody. Rescue experiments transfected Rab13HA cDNA into shRNA vector expressing cells followed by selection with both Puromycin and Zeocin.

### Three-dimensional culturing of DKO-1 cells in type-1 collagen

Cells were cultured in type-1 collagen as previously published^42^. In short, 500 DKO-1 cells were embedded in a 2.5 mg/mL type-1 collagen solution with 1 × DMEM and 10% FBS in a single wells (Advanced BioMatrix). Each well contained three separate 450 µL layers of collagen solution, with the cells embedded in the center layer. Each layer was solidified separately at 37 °C for 20–40 min. The collagen layers were covered with 500 µL DMEM media. When supplementing DKO-1 sEVs, each well was given ~ 10 µg of DKO-1 P100 sEVs every 4 days when the media was changed. Colonies were allowed to develop for 3 weeks at 37 °C, and then counted. A total of 9 different wells were counted per experiment and experimental condition.

### Transwell proliferation assays

Donor cells were seeded at a density of 0.05 × 10^6^ cells per Transwell. Recipient cells were seeded at a density of 0.1 × 10^6^ cells per well. Donor and recipient cells were seeded separately, washed three times in 1xDPBS, and then co-cultured in serum free medium. Following 48 h of co-culture at 37 °C, recipient cells were collected and then counted using a BioRad cell counter. Each experiment was carried out with three technical (individual wells) and three biological (distinct Transwell plates) replicates.

### Transwell luciferase reporter assays

Recipient cells were plated in 6-well plates at a density of ~ 2.5 × 10^5^ cells and cultured in DMEM supplemented with 10% bovine growth serum for 24 h. Media were replaced and cells were co-transfected (Promega, E2311, Madison, WI, USA) with 1.5 µg of Luc-reporter plasmid and 1.5 µg β-gal plasmid DNA/well. Donor cells were plated on top of 0.4 µm polyester membrane Transwell filters (Corning, 3450, Corning, NY, USA) at ~ 2.5 × 10^5^ cells/well for 24 h. Media from donor Transwells and recipient 6-well plates were removed and replaced with DMEM without FBS. Co-culture of donor and recipient cells was conducted for 48 h before recipient cells were harvested. Lysates were prepared in 1X Reporter lysis buffer (Promega, E2510) and luciferase assays were performed according to the manufacturers protocol (Promega, E2510). β-gal expression was simultaneously determined from the lysates (Promega). Differences in transfection efficiency were accounted for by normalizing Luc expression to β-Gal expression (Luc/β-Gal). All assays were performed on 3 biological replicates, each with 3 technical replicates.

### Proliferation assays

Cells were plated at 0.1 × 10^6^ cells per well in 12 well dishes (Corning). Individual wells were collected every 24 h following adherence, and cell counts were calculated using the BioRad Cell Counting System (BioRad). Each time point was collected three different times, and the mean was plotted to calculate a relative exponential growth curve (Prism 9).

### Soft agar assays

Soft Agar assays were carried out as described^9^. In short, 1% Noble Agar (Sigma-Aldrich) was warmed and then mixed with 2xDMEM media and 500 µL of the mixture was plated in each well and allowed to solidify at room temperature. 500 cells (1,500 cells per triplicate) were resuspended in 500 µL standard DMEM media and incubated with or without 10 µg DKO-1 P100 sEVs for 2 h at 37 °C. Following incubation, cells were centrifuged at 1000 rpm for 5 min (room temperature), and resuspended in 1.5 mL, 0.3% Noble Agar, 1 × DMEM solution followed by plating of 500 µL of the solution on top of the previous layer and allowed to solidify at room temperature until each layer solidified. 500 µL of fresh standard DMEM culture media was then added over the solidified cultures, supplemented with or without 10 µg of DKO-1 P100 sEVs. Cell media was then replaced every 4 days. Cultures were incubated at 37 °C for 2 weeks to allow colony formation. Colonies were stained with nitroblue tetrazolium chloride solution (Sigma) overnight at 37 °C, and then counted the following day.

### Immunocytochemistry

DKO-1 cells were seeded at 1 × 10^5^ cells per well in 12 well dishes (Corning) containing a single glass coverslip (Fisherbrand). Cells were given ~ 24 h to adhere and grow at 37 °C after which cells were washed twice in 1xPBS and fixed in 4% paraformaldehyde for 20 min at room temperature. Cells were permeabilized using 0.1% Triton X-100 for 15 min at room temperature and then incubated with 5% BSA and 5% donkey serum for 1 h at room temperature. Incubation with primary antibodies in 10% donkey serum at 1:100 dilution was overnight at 4 °C. Cells were then washed three times in 1% donkey serum for 10 min at room temperature, followed by incubation with secondary antibodies in 10% donkey serum at 1:100 dilution for 1 h at room temperature in the dark. Cells were then washed with 1% donkey serum in PBS three times for 10 min at room temperature before being mounted (VectaShield), sealed, and imaged on a Zeiss Observer Z.1.

### Transmission electron microscopy of EVs

TEM was carried out using a modified form of the protocol described previously^8^. Extracellular vesicles were purified by the above outlined high resolution iodixanol gradient and pooled into two populations: fractions 1–3 and fractions 4–6. These two pools were then absorbed onto carbon-coated formvar grids (Electron microscopy sciences) for 5 min at room temperature by floating the grids on 5 µL of the sample. After absorption of the vesicles, the grids were quickly washed in ddH2O, blotted, stained in 2% uranyl acetate for 30 s then dried on filter paper. Transmission electron microscopy was performed on a Tecnai T12 using an AMT CCD camera. Negative staining was performed in part through the use of the Vanderbilt Cell Imaging Shared Resource (supported by NIH grants CA68485, DK20593, DK58404, DK59637 and EY08126).

## Supplementary information

Supplementary file1
