# Supplementary material for: Rab13 regulates sEV secretion in mutant KRAS colorectal cancer cells
Source: Sci Rep. 2020 Sep 25;10:15804. doi: 10.1038/s41598-020-72503-8 (PMC7519665; doi:10.1038/s41598-020-72503-8)
Supplement: Supplementary file 1 — Supplementary file1 [file 41598_2020_72503_MOESM1_ESM.pdf]

Rab13 regulates sEV secretion in mutant *KRAS* colorectal cancer cells

Scott A. Hinger[1,5], Jessica J. Abner[1], Jeffrey L. Franklin[2], Dennis K. Jeppesen[3],

Robert J. Coffey[2,3,4], and James G. Patton[1,*]

Departments of Biological Sciences[1], Cell and Developmental Biology[2], Medicine[3], Vanderbilt University Medical Center, Nashville, TN, 37235, Veterans Affairs Medical Center[4], Nashville, TN 37235, and Vanderbilt University, Nashville, TN 37235, Present Address: Department of Physiology and Cell Biology, College of Medicine, The Ohio State University, Columbus, OH 43210.

Correspondence: james.g.patton@vanderbilt.edu

Supplemental Figure 1. NTA analysis of sEVs collected from DKO-1 cells under Rab13 knockdown conditions.

(A) Representative plot of vesicle size analyzed by NTA analysis of DKO-1 cells expressing an empty shRNA vector. (B) Representative plot of vesicle size analyzed by NTA analysis of DKO-1 cells expressing a scrambled shRNA vector. (C) Representative plot of vesicle size analyzed by NTA analysis of DKO-1 cells expressing shRNA #1 against Rab13. (D) Representative plot of vesicle size analyzed by NTA analysis of DKO-1 cells expressing shRNA #2 against Rab13.

Supplemental Figure 2. Proliferation of cells under Rab13 knockdown conditions.

(A) DKO-1 and DKs-8 cells under Rab13 knockdown conditions were seeded in 12 well plates and cultured for 120 hours. Cells were collected every 24 hours and counted. Data are from three biological replicates. Student's t-test found no significant difference between empty or shRNA-transfected cells.

Supplemental Figure 3. Transwell Assay Schematic.

(A) Transwell assays. Donor and recipient cells were seeded and grown separately for 24-48 hours before co-culture opposite a 0.4μm polyester membrane. (B) Proliferation assays using DKs-8 cells as donor cells with either normal levels of Rab13 or after Rab13 knockdown. Significance was determined by one way ANOVA. Data represent mean +/- SE, n=3. ns=no significance.

Supplemental Figure 4. Rab13 regulates growth in type-1 collagen via sEV secretion.

(A) Image of DKO-1 migratory colony morphology grown in type-1 collagen. (B) DKO-1 cells under Rab13 knockdown conditions were embedded in type-1 collagen with or without exposure to sEVs purified from DKO-1 cells. Migratory colonies were then counted under each condition.

(C) Quantitation of migratory colony counts under Rab13 knockdown conditions. Each data point represents a technical replicate from one of three biological replicates. Statistics were analyzed by one-way ANOVA. ** = $p < 0.01$, **** = $p < 0.0001$. ns=no significance.

Supplemental Figure 5. sEVs from Rab13 knockdown cells rescue colony growth in soft agar.

(A) DKO-1 cells with or without Rab13 knockdown were grown in soft agar for 2 weeks in the presence or absence of sEVs purified from Rab13 knockdown cells. (B) Quantification of colony counts from soft agar assay replicates. Significance was determined by one-way ANOVA. ** = $p<0.01$, **** = $p<0.0001$. Data represent mean +/- SE, n=3. ns = no significance.

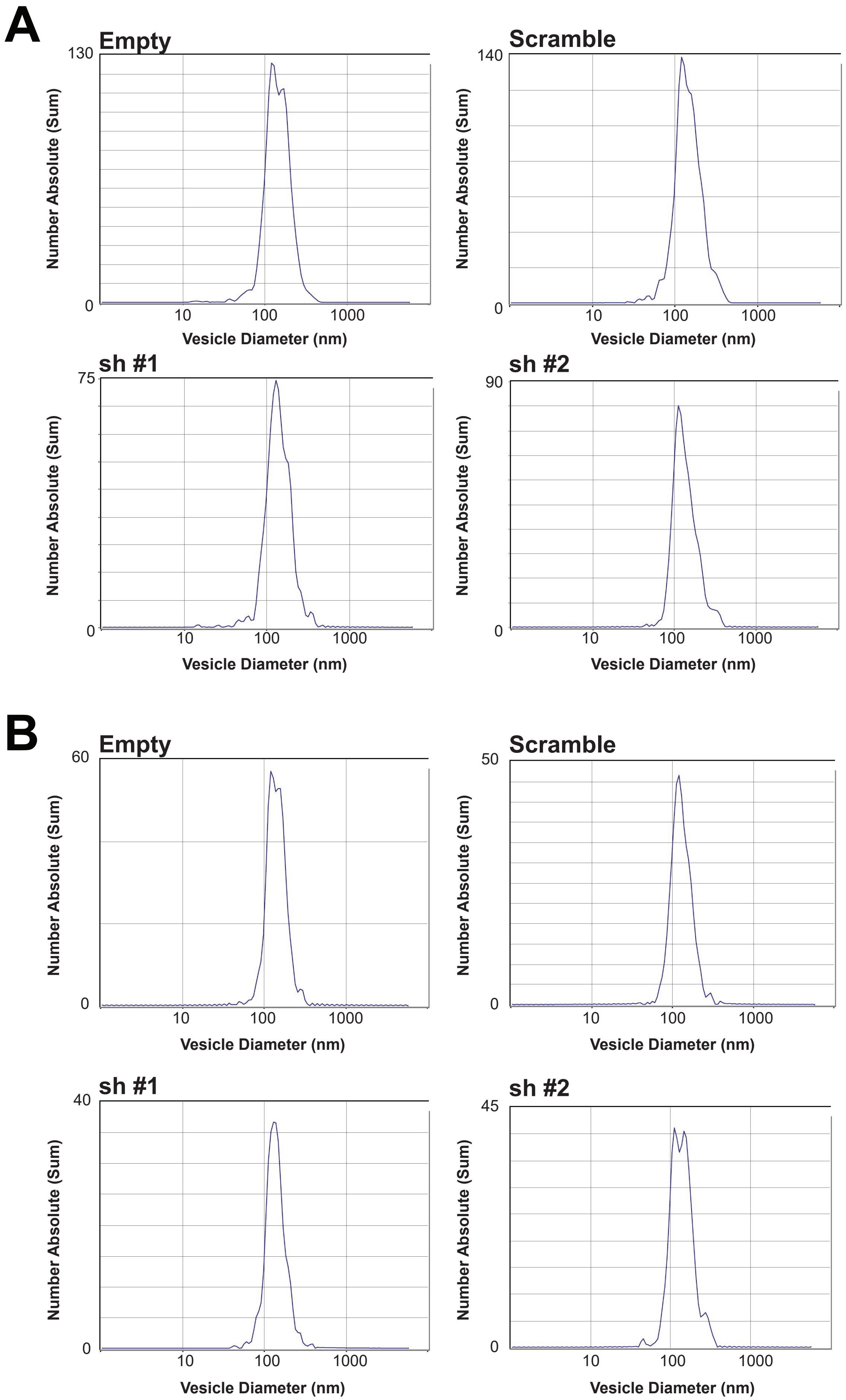
A
Empty
130
0
Number Absolute (Sum)
10
100
1000
Vesicle Diameter (nm)
Scramble
140
0
Number Absolute (Sum)
10
100
1000
Vesicle Diameter (nm)
sh #1
75
0
Number Absolute (Sum)
10
100
1000
Vesicle Diameter (nm)
sh #2
90
0
Number Absolute (Sum)
10
100
1000
Vesicle Diameter (nm)
B
Empty
60
0
Number Absolute (Sum)
10
100
1000
Vesicle Diameter (nm)
Scramble
50
0
Number Absolute (Sum)
10
100
1000
Vesicle Diameter (nm)
sh #1
40
0
Number Absolute (Sum)
10
100
1000
Vesicle Diameter (nm)
sh #2
45
0
Number Absolute (Sum)
10
100
1000
Vesicle Diameter (nm)

A

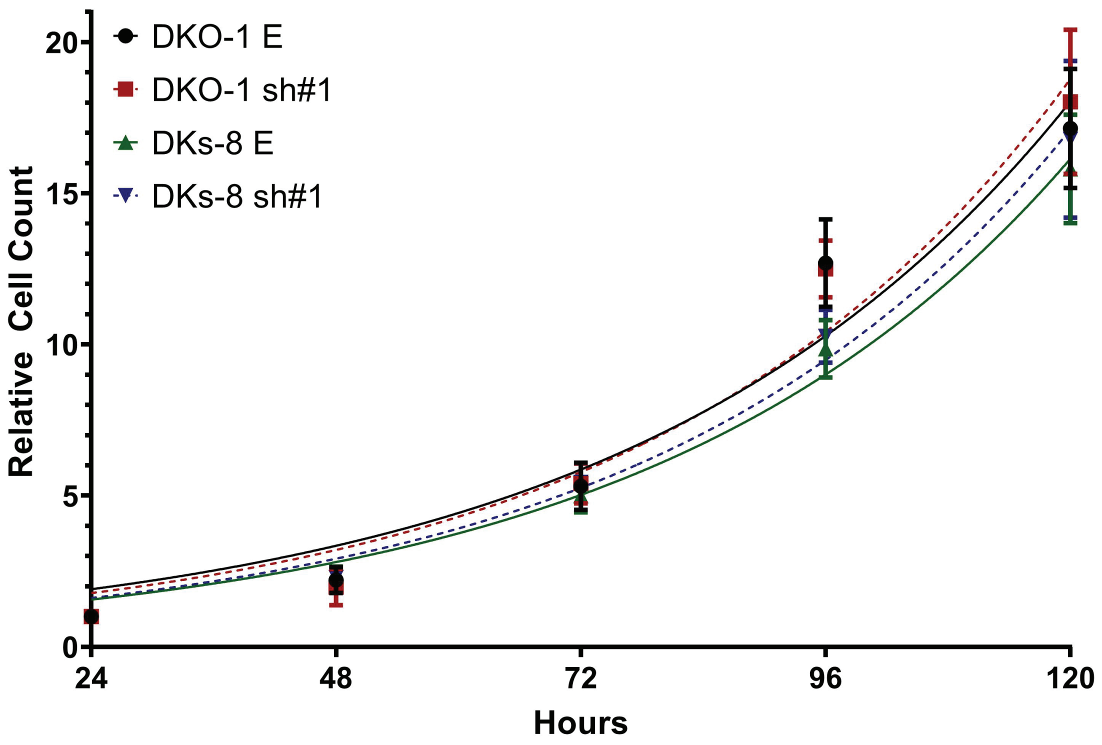

DKO-1 E
DKO-1 sh#1
DKs-8 E
DKs-8 sh#1
Relative Cell Count
Hours
0
5
10
15
20
24
48
72
96
120

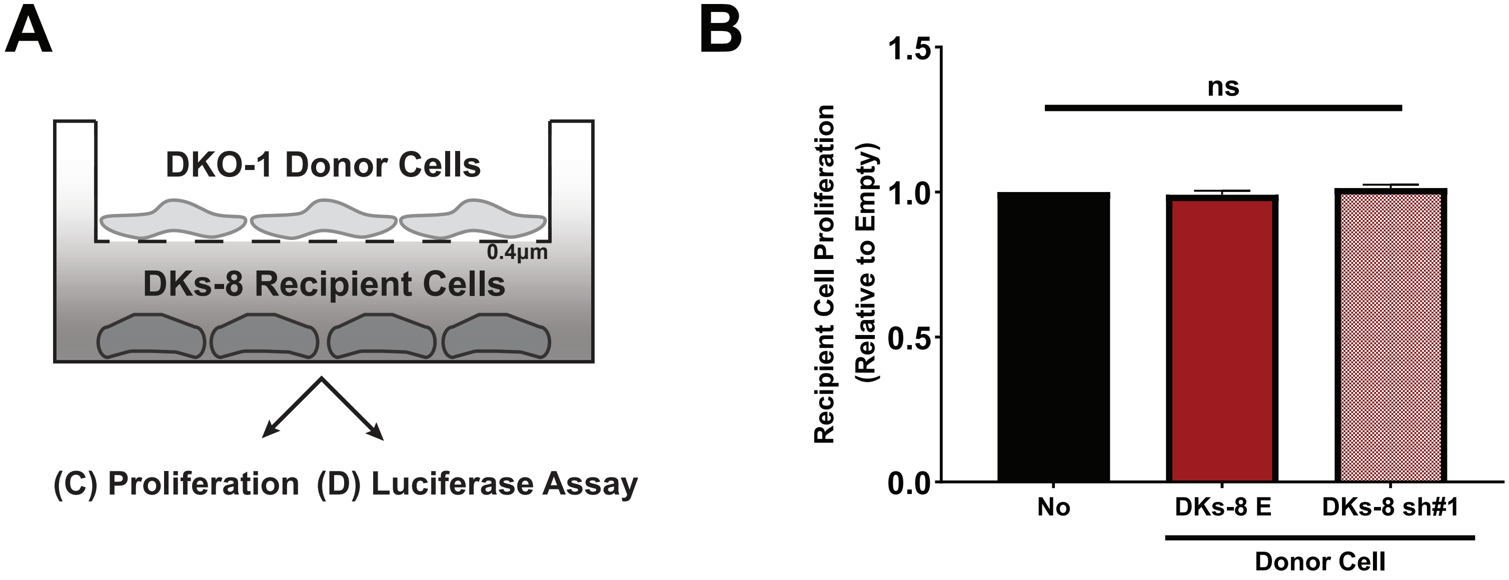
A
DKO-1 Donor Cells
0.4μm
DKs-8 Recipient Cells
(C) Proliferation
(D) Luciferase Assay
B
Recipient Cell Proliferation (Relative to Empty)
1.5
1.0
0.5
0.0
ns
No
DKs-8 E
DKs-8 sh#1
Donor Cell

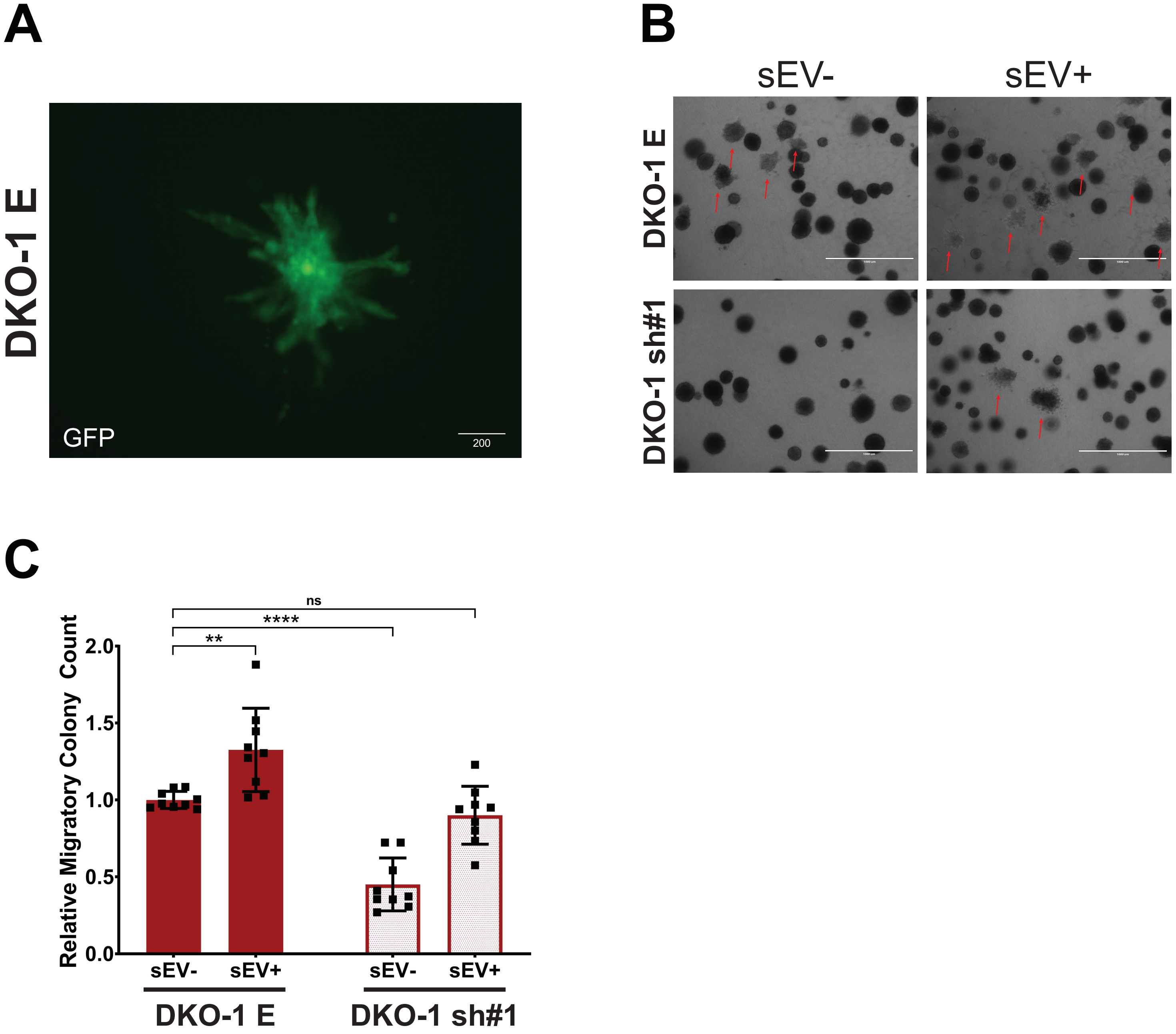
A
DKO-1 E
GFP
200
B
sEV-
sEV+
DKO-1 E
DKO-1 sh#1
C
Relative Migratory Colony Count
2.0
1.5
1.0
0.5
0.0
ns
****
**
sEV-
sEV+
sEV-
sEV+
DKO-1 E
DKO-1 sh#1

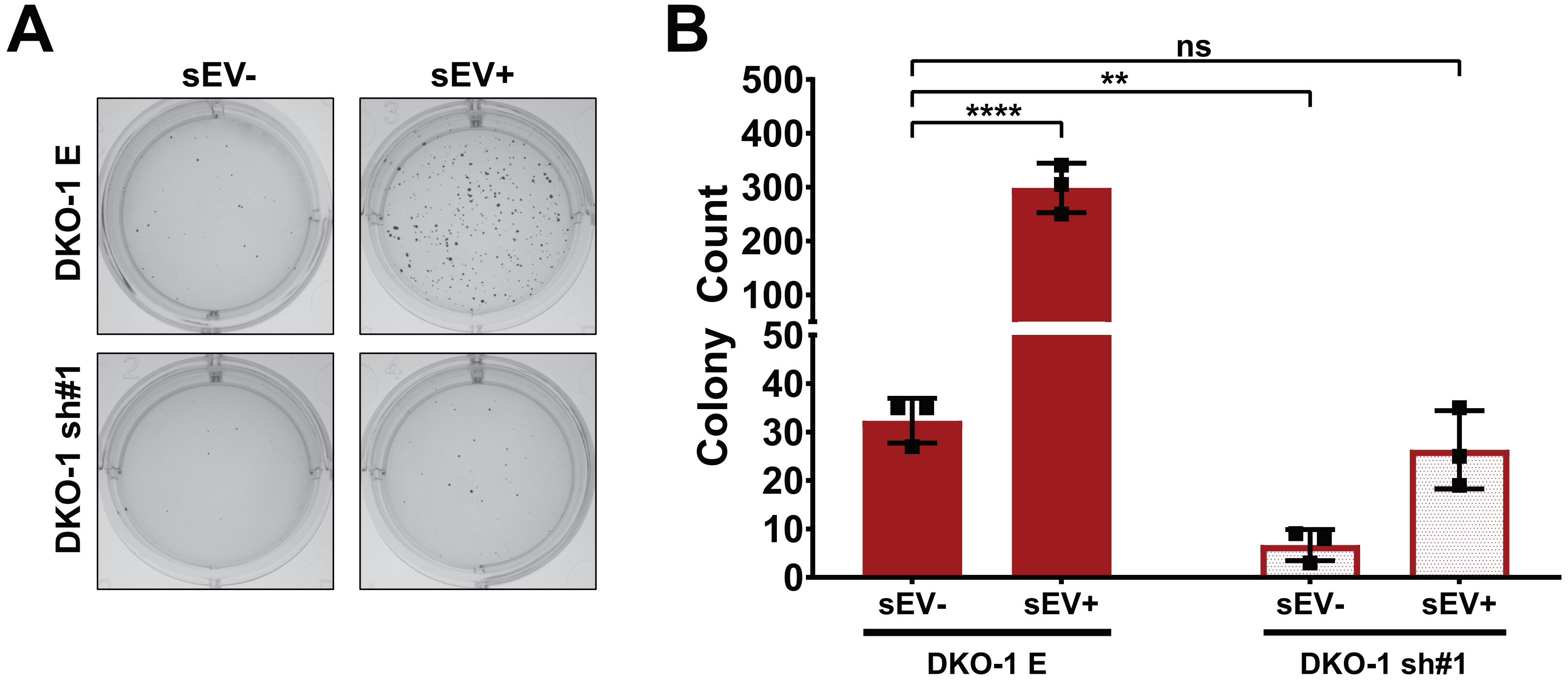
A
sEV-
sEV+
DKO-1 E
DKO-1 sh#1
B
ns
**
****
Colony Count
500
400
300
200
100
50
40
30
20
10
0
sEV-
sEV+
sEV-
sEV+
DKO-1 E
DKO-1 sh#1
